# Prior exercise and antioxidant supplementation: effect on oxidative stress and muscle injury

**DOI:** 10.1186/1550-2783-4-9

**Published:** 2007-10-03

**Authors:** Richard J Bloomer, Michael J Falvo, Brian K Schilling, Webb A Smith

**Affiliations:** 1 Department of Health and Sport Sciences, University of Memphis, Memphis, TN, USA

## Abstract

**Background:**

Both acute bouts of prior exercise (preconditioning) and antioxidant nutrients have been used in an attempt to attenuate muscle injury or oxidative stress in response to resistance exercise. However, most studies have focused on untrained participants rather than on athletes. The purpose of this work was to determine the independent and combined effects of antioxidant supplementation (vitamin C + mixed tocopherols/tocotrienols) and prior eccentric exercise in attenuating markers of skeletal muscle injury and oxidative stress in resistance trained men.

**Methods:**

Thirty-six men were randomly assigned to: no prior exercise + placebo; no prior exercise + antioxidant; prior exercise + placebo; prior exercise + antioxidant. Markers of muscle/cell injury (muscle performance, muscle soreness, C-reactive protein, and creatine kinase activity), as well as oxidative stress (blood protein carbonyls and peroxides), were measured before and through 48 hours of exercise recovery.

**Results:**

No group by time interactions were noted for any variable (P > 0.05). Time main effects were noted for creatine kinase activity, muscle soreness, maximal isometric force and peak velocity (P < 0.0001). Protein carbonyls and peroxides were relatively unaffected by exercise.

**Conclusion:**

There appears to be no independent or combined effect of a prior bout of eccentric exercise or antioxidant supplementation as used here on markers of muscle injury in resistance trained men. Moreover, eccentric exercise as used in the present study results in minimal blood oxidative stress in resistance trained men. Hence, antioxidant supplementation for the purpose of minimizing blood oxidative stress in relation to eccentric exercise appears unnecessary in this population.

## Introduction

Strenuous physical work, often novel and involving heavy resistance exercise inclusive of eccentric (i.e. lengthening) actions, causes muscle damage in a fiber-specific manner [1]. The initial mechanical insult produces damage that is evident within minutes [2] and displays sporadically throughout individual fibers (i.e. focal injury). This early damage can promote biochemical changes within the affected area, leading to generation of inflammatory cytokines and reactive oxygen species (ROS) that may further degrade muscle proteins and contribute to signs and symptoms of injury (e.g. loss of muscle force, muscle soreness, leakage of cellular proteins into the blood). Elevated oxidative stress biomarkers have been reported following strenuous anaerobic exercise in several investigations as we have previously reviewed in detail [3].

Both the performance of prior exercise and the use of antioxidant micronutrients have been used to attenuate the injury resulting from strenuous resistance exercise [4]. Prior exercise has been used to "precondition" the muscle and has been reported with great success in the literature as evidenced by significant reductions in structural or functional impairment [4], as well as alterations in immune response and protein turnover. Such adaptations appear evident for periods of weeks to months in untrained subjects [5]. This preconditioning of skeletal muscle is termed the "repeated bout effect" or "rapid adaptation" and has been reviewed in detail by McHugh [4]. However, to our knowledge, no study has investigated a potential "specific" repeated bout effect in men who currently resistance train, when exposed to a novel exercise stimulus. Such an unusual stimulus might include pure eccentric resistance exercise (which is difficult for individual trainees to perform on their own), in which subjects are subjected to repeated repetitions at higher loads than they might be able to handle for mixed concentric/eccentric exercise. Moreover, no study has measured oxidative stress biomarkers following heavy eccentric resistance exercise in subjects first exposed to preconditioning eccentric exercise. Therefore, this was of interest in the present study.

Because ROS may play a role in both the initiation and the progression of muscle fiber injury [3,6], it is hypothesized that antioxidants may function to minimize the extent of injury owing to their role in combating ROS. However, the use of antioxidants to attenuate exercise-induced muscle injury and oxidative stress has been met with mixed results, in particular with regard to resistance exercise. While some reports suggest a potential beneficial role of antioxidant supplementation in relation to a bout of resistance exercise [7-13], others indicate no benefit [14-20]. Discrepancies in findings may be due to the type, dosage, and timing of administration of the antioxidants, in addition to the exercise stress and the specific population being studied. In relation to the subject population in previous studies, with one exception [11] all studies reporting a benefit of antioxidant supplementation in attenuating muscle injury and oxidative stress following resistance exercise have included sedentary, non-resistance trained subjects. Therefore, while it is certainly possible that antioxidant supplementation may prove beneficial in resistance trained subjects, this is not well described in the literature. Endogenous antioxidant defenses may be heightened in individuals who regularly resistance train [21] and these individuals may not benefit greatly from exogenous antioxidant intake for purposes of attenuating signs and symptoms of muscle injury. With the widespread recommendations for use of antioxidant supplementation in the mainstream media, as well as in recreational and competitive athletics for purposes of reducing muscle injury and oxidative stress, an investigation focused on a population of resistance trained subjects was needed.

For these reasons, the purpose of the present investigation was to determine the independent and combined effectiveness of antioxidant supplementation and a prior bout of eccentric exercise in attenuating signs and symptoms of muscle injury, as well as oxidative stress biomarkers, in a population of resistance trained men. We hypothesized that muscle injury and oxidative stress would be elevated following strenuous eccentric exercise in all subjects, the extent of these changes would be less for subjects performing a prior bout of eccentric preconditioning exercise, and antioxidant supplementation would attenuate the degree of change in our sample of resistance trained men.

## Methods

### Subjects

Thirty-six healthy men volunteered to participate in this investigation. Subjects completed a medical history, diet and supplementation history, and physical activity questionnaire to determine eligibility. No subject was a vegetarian or a smoker, nor did they use tobacco products, anti-inflammatory drugs, or antioxidant supplements before (for a minimum of six months) or during the study period. Eligible subjects were those capable of concentrically bench pressing a load ≥ to their body mass, who performed resistance training using dynamic (concentric/eccentric) muscle actions for a minimum of one year before study participation (with no layoffs during this time period), and who performed upper-body resistance exercises at least once per week during the previous year. Characteristics of subjects who completed the study are provided in Table 1. All experimental procedures were performed in accordance with the Helsinki Declaration and the policy statement of the American College of Sports Medicine on research with human subjects. The University of Memphis Human Subjects Committee approved all experimental procedures. All subjects provided both verbal and written consent prior to participating in this investigation.

**Table 1:** Characteristics of 30 resistance trained men.

**Variable**	**No Prior Exercise Placebo**	**No Prior Exercise Antioxidant**	**Prior Exercise Placebo**	**Prior Exercise Antioxidant**
Age (yrs)	25 ± 5	23 ± 2	25 ± 4	22 ± 2
Height (cm)	177 ± 5	179 ± 2	176 ± 7	176 ± 3
Weight (kg)	92 ± 14	86 ± 11	92 ± 26	80 ± 13
Percent body fat	15 ± 5	11 ± 4	12 ± 6	10 ± 4
Bench Press 1RM (kg)	113 ± 19	116 ± 23	111 ± 31	99 ± 18
Resistance ex. (h·wk^-1^)	5 ± 2	4 ± 2	4 ± 2	4 ± 1
Resistance ex. (years training)	5 ± 3	6 ± 2	6 ± 3	4 ± 3

Data are mean ± SD. No significant differences between groups for any variable (P > 0.05).

### Outcome variables

Subjects were initially assigned to perform either a prior bout of eccentric exercise as described below, or no prior exercise. Within each of these groups, subjects were further assigned to receive either a placebo or antioxidant supplementation (1000 mg of vitamin C + 378 mg mixed tocopherols – 41 mg alpha, 3 mg beta, 84 mg delta, 250 mg gamma; and 39.5 mg mixed tocotrienols – 11 mg alpha, 1.5 mg beta, 5 mg delta, 22 mg gamma {FamilE™; Jarrow Formulas, Los Angeles, CA}). In both conditions, subjects consumed two capsules per day (one soft gel and one powder) to provide the above dosage, for 14 days prior to performing the eccentric exercise protocol as described below, and during two days of recovery. The use of the selected antioxidants, as well as the dosage and time of administration was based on our previous work demonstrating benefits in relation to muscle injury [7] and oxidative stress [9,22]. We have recently shown that the 14 day pretreatment period significantly elevates both plasma levels of vitamin C and vitamin E [22]. Both the soft gel (soybean oil) and powder (cellulose) placebos were identical in appearance to the antioxidants (packaged in typical one gram capsules). All group assignment was randomized and double-blinded. Following full randomization, nine subjects were assigned to one of four treatment groups: no prior exercise + placebo (NoP); no prior exercise + antioxidant (NoA); prior exercise + placebo (ExP); prior exercise + antioxidant (ExA).

### Experimental procedures

Subjects reported to the laboratory on several occasions as shown in Table 2. During visit one, anthropometric measurements were obtained, concentric bench press one-repetition maximum (1RM) was determined, and practice was provided for the isometric and dynamic muscle performance tests. For the 1RM testing, the ProSpot^® ^self spotting system was used, which "catches" the barbell at the top portion of the lift and lowers it back to the starting position, thereby negating the eccentric component. This system was used in order to avoid any additional familiarization associated with eccentric work. For 1RM testing, subjects warmed up with one set of 5 repetitions using 50% of their estimated 1RM and one set of 3 repetitions using 75% of their estimated 1RM, and then performed 3–5 single repetition sets until they attained their 1RM. An additional practice session was performed 48–72 hours after this initial visit. At each practice session for dynamic force, a total of four maximal concentric-only bench press throws, two each at 30% and 70%1RM, were performed. The 1RM testing as well as practice sessions did not induce any degree of muscle soreness in our sample of resistance trained men. Therefore, we do not believe that these sessions interfered with our outcome variables, as they might have if we had included previously non-resistance trained subjects. Following this second practice session, subjects received their group assignment. Those subjects not performing a prior bout of eccentric exercise started their assigned treatment for the 14 day period. Those subjects assigned to perform prior exercise (exactly as described below for the eccentric exercise protocol) were scheduled to do so within 48–72 hours following their second practice session. These subjects began their assigned treatment 72–96 hours following their prior exercise bout, for the 14 day period. During the 14 day pretreatment supplementation period, all subjects were instructed to resume their normal patterns of resistance training, with the exception of refraining from strenuous physical activity during the 48 hours preceding the eccentric exercise protocol. The 14 day period between the initial and second eccentric bout (for subjects in the prior exercise group) was chosen to allow for complete recovery from the initial bout, while allowing for any protection afforded by the preconditioning bout to remain present, as such adaptations have been reported to appear for periods up to months following the initial bout [5], at least for markers of muscle damage. There are presently no data in relation to attenuation of oxidative stress biomarkers and the repeated bout effect.

**Table 2:** Timeline to investigate the effects of prior exercise and antioxidants on muscle injury and oxidative stress.

**Group**	**Week 1**	**Week 2**	**Week 3**	**Week 4**	**Week 5**
*No Prior Exercise*	Paperwork, Baseline Measures & Familiarization	Supplementation	Supplementation	Eccentric Exercise Protocol (assessment pre, 0, 24, 48 hours post ex.)	
*Prior Exercise*	Paperwork, Baseline Measures & Familiarization	Prior bout of eccentric exercise	Supplementation	Supplementation	Eccentric Exercise Protocol (assessment pre, 0, 24, 48 hours post ex.)

Study Flow in Sequential Steps:

1. Baseline Assessment

Anthropometrics, 1RM, familiarization (Week 1)

2. Group Assignment

*No prior exercise*:

Start supplementation (Week 2)

*Prior exercise*:

Perform prior bout of eccentric exercise (Week 2)

Start supplementation (Week 3)

3. Eccentric Exercise Protocol

*No prior exercise*:

Week 4

*Prior exercise*:

Week 5

### Eccentric exercise protocol

During the two days preceding the eccentric exercise protocol subjects were asked to avoid strenuous physical activity. They reported to the lab in the morning following an eight hour fast. Subjects performed 10 sets of 10 repetitions of the barbell bench press exercise using 70% concentric 1RM. To ensure subject safety and for ease of administration, a Smith-machine was chosen for this exercise. The barbell was lowered for five seconds at which time it came in contact with their chest at the end of the five second period. At this time, spotters raised the barbell to the arms extended starting position (< 1 second) and the subject immediately began lowering the bar for the next repetition. This continued until all 10 repetitions were performed. If subjects were unable to lower the barbell under control for 5 seconds, the bar was secured, 10% of the total weight was removed, and the next repetition immediately resumed. Subjects were provided 120 seconds rest between each set and were provided water *ad libitum*. Through pilot testing, we found that the load and rest interval length used here were sufficient to induce considerable muscle soreness and loss of muscle force, and realistic to complete. That is, when using loads greater than 70%1RM, subjects could not complete all repetitions, even during the initial sets, requiring the weight to be reduced drastically on subsequent sets. Therefore, even though use of a protocol inclusive of greater loads may have induced more injury, and thus potentially a greater degree of adaptive protection, it simply was not realistic for subjects to perform.

The volume-load of the eccentric exercise protocol was compared between groups as calculated by the following equation: Volume-load = load (kg) × 9.81 (m/s^2^) × barbell displacement (m) × #repetitions × #sets. The actual load used during the protocol was used in this equation, accounting for any potential decrease in load due to fatigue. Rating of perceived exertion (RPE) using the 6–20 Borg scale and heart rate (Polar A5 monitors) was also assessed at the completion of each set and the average value over 10 sets was calculated.

### Dependent variables

The dependent variables in this investigation are commonly used in the study of exercise-induced muscle injury and oxidative stress [23]. The variables described below (with the exception of antioxidant reducing capacity – measured only pre exercise; and lactate – measured only pre and immediately after exercise) were measured pre, immediately after, 24 and 48 hours after the eccentric exercise protocol. The order of data collection was as follows.

### Bloodborne variables

For each time point, approximately 15 mL of blood was collected into vacutainer tubes via antecubetal venipuncture. The pre exercise sample was taken following a 10-minute quiet rest period. Blood from tubes containing EDTA was placed on ice and immediately used for analysis of whole blood lactate (Accutrend; Roche Diagnostics, Mannheim, Germany). The remainder of blood was immediately processed by centrifugation to obtain plasma. Blood collected into vacutainer tubes containing no additive was allowed to clot at room temperature for 30 minutes and then processed by centrifugation to obtain serum. Both plasma and serum samples were stored in multiple aliquots at -80°C until analyzed for markers described below. All assays were performed in duplicate on first thaw.

As a marker of sarcolemma disruption, serum creatine kinase activity was measured spectrophotometrically using commercially available reagents (StanBio Labs, Boerne, TX). The coefficient of variation for this assay was 4.1%. As a marker of systemic inflammation, serum C-reactive protein was measured using an ultra-sensitive sandwich ELISA procedure (Diagnostic Systems Laboratories, Inc., Webster, TX). The coefficient of variation for this assay was 5.1%. Plasma antioxidant reducing capacity (represented as uric acid equivalents) was determined as a marker of blood antioxidant capacity using commercially available reagents (Northwest Life Science Specialties, Vancouver, WA). The coefficient of variation for this assay was 6.3%. As markers of oxidative stress, plasma protein carbonyls were measured using an ELISA according to the procedures recommended by the manufacturer (Zentech Technology, Dunedin, New Zealand), and plasma total peroxides were measured using a colorimetric assay with hydrogen peroxide as the standard, as described by the reagent manufacturer (Pierce Biotechnology, Inc., Rockford, IL). The coefficient of variation for these assays was 3.8% and 4.7%, respectively. These two measures of oxidative stress were chosen as they represent oxidation of both proteins and lipids, possibly related to oxidized contractile and/or structural muscle proteins and lipid containing cell membranes. To ensure quality control, both high and low calibrated controls were used in each ELISA procedure.

### Muscle soreness

Using a 10 cm visual analog scale (where "0" represents no pain and "10" represents intense pain) subjects reported their perceived muscle soreness following the performance of two (concentric-eccentric) repetitions of the barbell bench press exercise using a standard 20 kg barbell. Subjects were familiarized to these procedures prior to the day of the eccentric protocol.

### Maximal isometric force

Maximal isometric force in a bench press position was measured using a customized force plate and power rack design. This power rack had one-inch hole spacing for individual bar height adjustments. The upper arm was fixed parallel to the floor with a 90° angle about the elbow joint and the bar was in line with the mid-sternum. From this position, the corresponding grip width and fixed bar height were recorded and reproduced for all testing sessions. Calibration of the force plate was performed daily according to manufacturer's recommendations (Rice Lake Weighing Systems; Rice Lake, WI). Subjects performed two submaximal contractions at 50% and 75% perceived effort, followed by two maximal voluntary contractions of three to five seconds in duration, interspaced with 30 seconds of rest. Subjects were given instruction to contract as hard and as fast as possible. Data were sampled at 1000 Hz and channeled though a 12-bit analog-to-digital converter (DAS1200Jr; Measurement Computing, Middleboro, MA). Data were smoothed using a 4^th^-order recursive Butterworth digital filter (cutoff frequency 30 Hz) and analyzed for peak force using Datapac 2k2 (v3.12; Mission Viejo, CA).

### Maximal dynamic force and peak velocity

Bench press throws were performed using a self-spotting device capable of supporting a standard size barbell (mass = 6 kg) via cables (ProSpot Fitness^®^, Norcross, GA). Two maximal attempts were performed using 30% 1RM interspaced with 30 seconds of rest. The movement began with the bar against the subjects' chest in line with the mid-sternum. When given the command from the investigator, subjects attempted to throw the bar for maximal height. Grip widths corresponding to those of the isometric test were maintained throughout testing. Subjects were also instructed to keep their entire body in contact with the bench and force plate. Kinetic and kinematic data were acquired through the combination of a modified force plate (as described above) and linear velocity/position transducer (VP510; Unimeasure; Corvallis, OR). Data were sampled as described previously, except that a 20 Hz cutoff frequency was used for velocity. For both the isometric and dynamic tests, the higher of two values for each measure was used in the analyses.

### Dietary records

All subjects were instructed to maintain their normal diet during the study period and completed daily food records for the two days before testing, the day of testing, and the day following testing. Records were analyzed for total kilocalories, protein, carbohydrate, fat, vitamin C, vitamin E, and vitamin A using commercially available software (Diet Analysis Plus, ESHA Research, Salem, OR).

### Statistical analysis

The data for all dependent variables were analyzed using a 4 (group) × 4 (time) repeated measures ANOVA. Significant interactions and main effects were analyzed using Tukey's *post hoc *test. As sample sizes were relatively small amongst groups, we performed the Kolmogorov-Smirnov and Mauchly tests to confirm normality and homogeneity of variances, respectively. From our dependent variables, we identified creatine kinase activity and C-reactive protein as those not following a normal distribution and were subsequently log transformed for analysis. The Mauchly test confirmed sphericity of our data. Subject characteristics, dietary variables, and antioxidant reducing capacity data were compared between subject groups using a one-way ANOVA. Pairwise correlations for all dependent variables were generated. Effect size calculations were performed using Cohen's D. All analyses were performed using JMP statistical software (SAS Institute, Cary, NC). Statistical significance was set at P ≤ 0.05. The data for dependent variables are presented as mean ± SEM. Subject characteristics, exercise related measures (e.g. work, RPE, heart rate, and lactate), and dietary data are presented as mean ± SD.

## Results

Of the 36 subjects who started the study, only data from 30 subjects (NoP, n = 8; NoA, n = 7; ExP, n = 7; ExA, n = 8) were included in the analysis. Characteristics of these 30 subjects are included in Table 1. Six subjects did not complete all aspects of the study due to personal reasons (e.g. lack of time, problems with blood donation, injury resulting from accident unrelated to study). Compliance to supplementation was ≥ 99% for all groups based on capsule counting upon return of bottles. No differences were observed between NoP, NoA, ExP, or ExA for total work (28474 ± 11470; 28434 ± 11724; 30515 ± 4981; 28622 ± 5943 kJ), RPE (16 ± 2; 18 ± 1; 17 ± 1; 16 ± 2), heart rate (140 ± 15; 143 ± 16; 137 ± 21; 132 ± 12 bpm), or blood lactate (7.8 ± 2.9; 9.5 ± 1.5; 7.5 ± 2; 9.3 ± 2.5 mmol·L^-1^), respectively (P > 0.05). The mean daily kilocalories, protein, carbohydrate, fat, vitamin C, vitamin E, and vitamin A intake during the study period did not differ between groups (P > 0.05). Dietary data are shown in Table 3.

**Table 3:** Dietary intake of resistance trained men before and following the eccentric exercise protocol.

**Group**	**Kcal**	**Protein**	**Carbohydrate**	**Fat**	**Vitamin C**	**Vitamin E**	**Vitamin A**
*No Prior Exercise Placebo*	2534 ± 444	153 ± 12	253 ± 73	94 ± 23	66 ± 39	11 ± 8	1441 ± 486
*No Prior Exercise Antioxidant*	2846 ± 926	161 ± 28	310 ± 79	102 ± 44	126 ± 55	9 ± 9	803 ± 581
*Prior Exercise Placebo*	2886 ± 604	155 ± 33	333 ± 99	97 ± 30	106 ± 59	7 ± 6	1993 ± 624
*Prior Exercise Antioxidant*	2540 ± 584	136 ± 51	282 ± 67	89 ± 36	107 ± 81	7 ± 5	1149 ± 497

Data are mean ± SD. No statistically significant differences were noted between groups for any measured variable (p > 0.05). Gram quantities for each macronutrient are provided. Vitamin C and vitamin E values are provided in mg; vitamin A values are provided in retinol equivalents.

### Bloodborne variables

**Figure 1:**
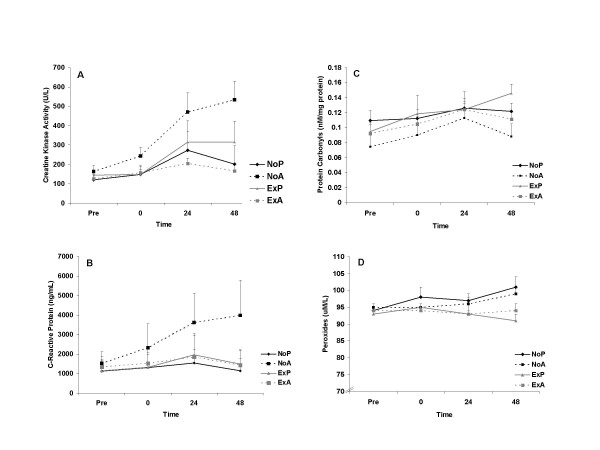
Creatine kinase activity (A), C-reactive protein (B), protein carbonyls (C) and peroxides (D) before and following an acute bout of eccentric resistance exercise in trained men. No prior exercise + placebo (NoP); no prior exercise + antioxidant (NoA); prior exercise + placebo (ExP); prior exercise + antioxidant (ExA). No significant group differences noted for any variables (P > 0.05). A time main effect was noted only for creatine kinase activity (P < 0.0001).

A time main effect was noted for creatine kinase activity (P < 0.0001) with values collectively peaking at 24 hours post exercise (317 ± 29U·L^-1^) compared to pre exercise (139 ± 29U·L^-1^). Creatine kinase activity was significantly higher than pre exercise at 24 and 48 hours post exercise (P < 0.05). Creatine kinase activity appeared lower in the prior exercise groups, however no statistical interaction was noted (P = 0.252). C-reactive protein was not different between groups (P = 0.091) and collectively was elevated to the greatest extent at 24 hours post exercise with no time main effect noted (P > 0.05). Neither creatine kinase activity nor C-reactive protein followed a normal distribution and therefore, data were log transformed prior to analysis. However, for ease of comparison to other published studies using these biomarkers, the original values are included in Figure 1. Pre exercise antioxidant reducing capacity was not different (P = 0.422) between NoP, NoA, ExP, or ExA (0.206 ± 0.067; 0.265 ± 0.071, 0.336 ± 0.077; 0.354 ± 0.063 mmol·L^-1^), respectively. Protein carbonyls were not statistically different between groups (P = 0.351), but experienced a peak from pre to 24–48 hours post exercise that failed to reach significance (P = 0.152). Creatine kinase activity was correlated to both protein carbonyls (r = 0.391, P < 0.00001) and C-reactive protein (r = 0.403, P < 0.00001). No statistical main effects or interaction were noted for peroxides (P > 0.05). Data for bloodborne variables are shown in Figure 1.

### Muscle soreness and function

**Figure 2:**
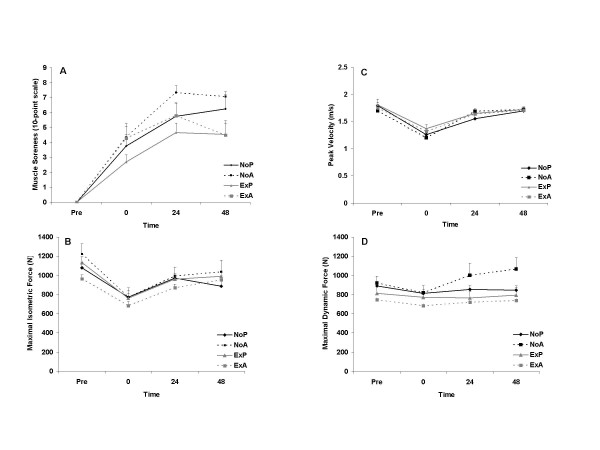
Muscle soreness (A), maximal isometric force (B), peak velocity (C), and maximal dynamic force (D) before and following an acute bout of eccentric resistance exercise in trained men. No prior exercise + placebo (NoP); no prior exercise + antioxidant (NoA); prior exercise + placebo (ExP); prior exercise + antioxidant (ExA). No significant group differences noted for any variables (P > 0.05). Time main effects were noted for muscle soreness, maximal isometric force, and peak velocity (P < 0.0001).

A time main effect was noted for muscle soreness (P < 0.0001) with values peaking 24 hours post exercise (5.9 ± 0.3/10-point scale), and elevated above pre exercise at all times post exercise (P < 0.05). No interaction effect was noted for muscle soreness (P = 0.324). Muscle soreness was significantly correlated to creatine kinase activity (r = 0.288, P < 0.0001), but the relationship is weak. Baseline muscle performance variables were not different between groups (P > 0.05). Time main effects were also noted for maximal isometric force and peak velocity (P < 0.0001), with lower values immediately and 24 hours post exercise compared to pre exercise (P < 0.05). Values remained below pre exercise at 48 hours post exercise for maximal isometric force but recovered by 24 hours post exercise for peak velocity. The pattern of change for these variables was not different between groups, evidenced by insignificant interaction effects for both isometric force (P = 0.742) and peak velocity (P = 0.847). Peak dynamic force was decreased slightly immediately post exercise, but insignificantly (P = 0.082), and recovered to pre exercise values by 24 hours post exercise. No interaction effect was noted for peak dynamic force (P = 0.563). Data for muscle soreness and function are shown in Figure 2.

Effect size calculations performed using Cohen's D indicated only small effect sizes (<0.4) for most measured variables at most times following exercise. The only exceptions to the finding of small effect sizes occurred for creatine kinase activity and C-reactive protein at both 24 and 48 hours post exercise, where effect size calculations ranged from 0.46–0.87, indicating small to moderate effects.

## Discussion

The findings of the present study partially refute our initial hypotheses. That is, no effect was observed for the combination of prior exercise and antioxidant supplementation in our sample of resistance trained subjects. Based on these data, there appears to be no independent or combined effect of prior exercise or antioxidant supplementation as provided here on markers of muscle injury or oxidative stress following muscle damage-inducing exercise.

Due to the novelty of our chosen exercise model we initially believed that the performance of a preconditioning session would allow for an adaptation in accordance with the repeated bout effect, which is quite evident for non-resistance trained subjects, as described by McHugh [4]. Despite the fact that our subjects were resistance trained, no individual used eccentric exercise exclusively, especially using the high volume-load employed in the present study. Therefore, we believed that the novelty of such a prior bout would lend protection against the subsequent bout in relation to muscle injury and oxidative stress. However, this was not the case, and we need to reject our initial hypothesis. While creatine kinase activity, muscle soreness, maximal isometric force and peak velocity were altered in response to the exercise bout, indicative of a muscle injury response, there were no group differences noted for any variable. It should be noted however, that the no prior exercise + antioxidant group demonstrated a large (albeit nonsignificant) rise in both creatine kinase activity and C-reactive protein at all times post exercise. It is possible that a pro-oxidant effect was apparent in this group, although other markers did not appear to differ greatly between this group and the others. Further study would be needed to address this hypothesis.

We have come up with some possible explanations in relation to our findings. First, near maximal adaptations associated with the repeated bout effect are already present in resistance trained men. Therefore, additional adaptations resulting from a novel stimulus such as pure eccentric exercise are minimal. It is also possible that our chosen exercise, despite the use of pure eccentric actions, was not unique enough to subjects. In relation to the above, while we do not believe this played a major role in the present study, it is possible that our initial 1RM and familiarization testing sessions could have provided a slight degree of adaptive protection to subjects in the no prior exercise group. If so, this would have masked group differences in our dependent measures. Second, it is possible that the exercise stress used in the present investigation was not strenuous enough to induce significant muscle damage, potentially disallowing for any further repeated bout effect to be noted in our already resistance trained subjects. Therefore, any small group differences were masked by the small change in the variables measured. However, based on our findings for elevated muscle soreness and creatine kinase activity, as well as impaired muscle function, which are similar to the magnitude and pattern of change presented in other investigations using trained subjects [16,24,25], we do not believe this second explanation to be the case. Related to this, it should be noted that the large degree of variability in subjects' responses in both CK activity and CRP also interfered with our ability to detect group differences. Finally, our relatively small sample size, indeed decreased our statistical power to detect group differences, and may have resulted in a type II error, especially considering the subject to subject variability in some of our measures. However, effect size calculations were performed using Cohen's D and we noted only small effect sizes (<0.4) for all measured variables at times following exercise, with the exception of creatine kinase activity and C-reactive protein at 24 and 48 hours post exercise (effect sizes for these two variables ranged from 0.46–0.87, indicating small to moderate effects). This underscores our findings in the main model of no significant group differences for any measured variable. However, we admit that our small sample size is a limitation of the present work and future studies using larger sample sizes are needed to confirm these findings. Future studies should take note of our post-hoc power analyses in which we identify a sample size of 44 total subjects (n = 11 subjects per group) in order to achieve a statistically detected interaction effect for C-reactive protein, 62 total subjects for creatine kinase activity, 42 total subjects for peroxides, 52 total subjects for protein carbonyls, 122 total subjects for maximal isometric force, and 144 total subjects for peak velocity. This assumes similar variance and effect as we observed in our sample of resistance trained subjects.

Oxidative stress biomarkers in the present study were relatively unaffected by exercise or treatment. While several studies have reported increased oxidative stress following pure eccentric and mixed eccentric/concentric resistance exercise as reviewed previously [3], the majority have used untrained individuals as research participants. We have recently reported a minor increase [25], as well as no change [24] in both protein carbonyls and malondialdehyde using highly resistance trained subjects. In agreement with our recent findings, Ramel et al. [26] reported a lower response in lipid peroxidation products (e.g. conjugated dienes) in trained versus untrained subjects following resistance exercise. Additionally, McAnulty et al. [27] reported no change in F_2_-isoprostanes or plasma antioxidant potential in strength trained subjects following a two-hour resistance training workout. Taken together, it appears that resistance trained subjects experience minimal change in blood oxidative stress biomarkers (at least as measured by lipid and protein oxidation) following strenuous resistance exercise, during the 48 hour recover period. This may be due to heightened antioxidant defense mechanisms as a result of chronic resistance exercise exposure as reviewed previously [3]. This is an important finding that merits attention, especially considering the elevated concern by some over radical species related to acute exercise. Despite these findings, it should be noted that we did not measure muscle tissue oxidative stress in our subjects. It is possible that muscle oxidative stress could have been present despite our findings of minimal change in blood markers of oxidative stress. Moreover, we only included the measurement of peroxides and protein carbonyls as our chosen oxidative stress variables. It is possible that other biomarkers of oxidative stress may have experienced a different response. Lastly, our sampling time was limited to 48 hours post exercise, and it is possible that further changes in our measured variables may have been present at times latter into recovery. These are indeed limitations of the present study.

Antioxidant supplementation had no impact on our chosen dependent variables. Prior studies have reported varying degrees of protection against muscle soreness and/or strength loss [7,8,10,11,13], inflammation [12], and oxidative stress [9,11] following antioxidant supplementation. However, only McBride et al. [11], providing 1200 IU per day of vitamin E for 14 days before exercise, used resistance trained subjects. Many other investigations report little to no benefit of antioxidant supplementation in relation to muscle injury or oxidative stress [14-20]. Of these studies, only Bloomer et al. [16] used resistance trained men as subjects, supplementing the carotenoid astaxanthin for three weeks before eccentric resistance exercise. It should be noted that individuals who regularly resistance train likely have heightened endogenous antioxidant defense mechanisms [21]. Because of such adaptations, these individuals may not benefit from further exogenous antioxidant intake for purposes of attenuating signs and symptoms of muscle injury. Although, this likely depends on which component of the endogenous antioxidant defense system is favorably altered by exercise training. Based on data from the present study, this appears to be the case. However, this does not mean that the antioxidants provided in the present study would not prove beneficial for other purposes (e.g., general health). Nor do these findings indicate that all antioxidants would yield no effect in regards to the markers assessed in the present investigation. From a training adaptation standpoint, it is uncertain as to whether or not exogenous antioxidant supplementation may decrease the adaptive response to resistance exercise. This is an area of study that requires further attention.

It is important to note that due to the potential effect of confounding variables in the present investigation (e.g., prior training status of subjects), our ability to detect significant differences in the measured variables may have been affected. Therefore, we are uncertain whether or not the antioxidant and/or prior exercise was simply ineffective or whether or not confounding variables interfered with our ability to detect group differences. Future study is needed in this area before firm recommendations can be made regarding the use of antioxidant supplements for purposes of attenuating exercise-induced muscle injury and oxidative stress in resistance trained individuals.

Our antioxidant selection, as well as the dosage and timing of administration, was based on our previous work demonstrating benefits in relation to both oxidative stress [9,22] and muscle injury [7]. However, these previous findings are in reference to untrained women [7,9] following eccentric resistance exercise, and aerobically trained subjects following strenuous endurance exercise [22]. It is very possible that eccentric resistance exercise induces a different response in ROS than does aerobic work (e.g. neutrophil respiratory burst activity, ischemia-reperfusion, etc. versus electron leakage through the respiratory chain). Therefore, caution needs to be used when extrapolating findings across different exercise modes, as well as across subject populations. Moreover, because we ceased measurements at 48 hours post exercise, it is unknown what the pattern of response for the dependent variables would have been during the days following. Future studies may choose to investigate the longer term time course of change in these variables following treatment as used here.

## Conclusion

We report no benefit of preconditioning eccentric exercise or antioxidant supplementation on attenuating markers of muscle injury or oxidative stress in resistance trained men. It is possible that these individuals already have adequate protection, both structurally and biochemically, resulting from chronic resistance exercise exposure. Additional support from exogenous antioxidants as provided in the present study, and/or prior novel exercise appears unnecessary. Therefore, under the current experimental constraints, our findings do not support the use of the specific antioxidant supplements for purposes of decreasing resistance exercise-induced muscle damage or oxidative stress in those already considered to be well-trained.

## Competing interests

The author(s) declare that they have no competing interests.

## Authors' contributions

RB was responsible for the study design, biochemical work, statistical analyses, and manuscript preparation; MF was responsible for the study design and all data collection; BS was responsible for the study design and analysis of all performance related variables; WS was responsible for blood collection, blood processing and biochemical work. All authors read and approved of the final manuscript.
